# Effect of disease duration on fecal biomarkers in ulcerative colitis: a prospective cohort study

**DOI:** 10.1186/s12876-022-02502-8

**Published:** 2022-09-15

**Authors:** Natsuki Ishida, Masanao Kaneko, Yusuke Asai, Takahiro Miyazu, Satoshi Tamura, Shinya Tani, Mihoko Yamade, Moriya Iwaizumi, Yasushi Hamaya, Satoshi Osawa, Takahisa Furuta, Ken Sugimoto

**Affiliations:** 1 First Department of Medicine, Hamamatsu University School of Medicine, 1-20-1 Handayama, Higashi-ku, Hamamatsu, Shizuoka 431-3192 Japan; 2 Department of Endoscopic and Photodynamic Medicine, Hamamatsu University School of Medicine, Hamamatsu, Shizuoka Japan; 3 Department of Laboratory Medicine, Hamamatsu University School of Medicine, Hamamatsu, Shizuoka Japan; 4 Center for Clinical Research, Hamamatsu University School of Medicine, Hamamatsu, Shizuoka Japan

**Keywords:** Ulcerative colitis, Fecal calprotectin, Fecal immunochemical occult blood test, Biomarker, Disease duration

## Abstract

**Background:**

Biomarkers such as fecal calprotectin (FC) and fecal immunochemical occult blood tests (FIT) for ulcerative colitis (UC) are used in clinical practice. In this study, the effect of UC disease duration on FC was investigated and compared to that on FIT.

**Methods:**

One hundred twenty-eight colonoscopic examinations and two fecal biomarkers measurements were performed. The cases of UC were divided into short- and long-term disease-duration groups or categorized into three groups with disease durations of 0–5, 6–13, and 14–38 years. We analyzed correlations between biomarker levels and endoscopic scores, including the Mayo endoscopic subscore (MES), ulcerative colitis endoscopic index of severity, and the sum of MES.

**Results:**

In the analysis of short- and long-term disease durations, the three endoscopic scores and biomarker levels showed significant correlations in both long-term and short-term groups. Most of the correlation coefficients for the individual long-term group were lower than the corresponding values for all cases, while most of the correlation coefficients for the individual short-term groups were higher than the corresponding values for all cases. In the three-group analysis (disease durations of 0–5, 6–13, and 14–38 years), the two biomarkers and three endoscopic scores showed significant correlations, and most of the correlation coefficients between biomarkers and endoscopic scores tended to be lower in the long-term follow-up group. In the receiver operating characteristic analysis for predicting mucosal healing in the three groups, the area under the curve for FC and FIT concentrations in the 0–5 year disease-duration group showed particularly higher values than those for the other two groups.

**Conclusions:**

Similar to FIT, FC is affected by the duration of UC, indicating that FC may be a highly useful biomarker, especially in short-term disease.

## Background

Ulcerative colitis (UC) is a refractory disease characterized by symptoms such as diarrhea, bloody stools, and abdominal pain with repeated relapses and remissions [1]. Evaluation of UC activity is important, and endoscopic evaluations of mucosal inflammation can facilitate subsequent prognostication [2, 3]. On the basis of these considerations, achievement of mucosal healing is important in inflammatory bowel disease (IBD), and evaluation of IBD using endoscopy is extremely important. However, endoscopic examinations are associated with problems such as physical burden to the patient, cost, and risk of complications.

Thus, to avoid the need for frequent endoscopy, noninvasive biomarkers that accurately reflect the endoscopic activity of UC have emerged, and fecal calprotectin (FC) and the fecal immunochemical occult blood test (FIT) are being widely used in clinical practice [4–9]. FC is a calcium-binding protein found in neutrophils, monocytes, and macrophages, accounting for approximately 60% of the cytoplasm [10]. When inflammation occurs locally in the intestinal tract, leukocytes, including neutrophils, migrate into the lumen through the intestinal wall; thus, the inflammatory state of the intestinal tract can be determined by measuring the amount of calprotectin in feces [11]. The FIT was originally developed for colorectal cancer screening, and in UC, it reflects bleeding caused by inflammation of the mucosa and has been reported to correlate with the endoscopic score [6]. FC and FIT have been reported to not only reflect endoscopic activity but also predict subsequent relapse in patients with UC in clinical remission [12–15]. In addition to these fecal biomarkers, serum leucine-rich alpha 2 glycoprotein (LRG) has been reported to be a useful marker that reflects endoscopic activity in UC and has been used in clinical practice [16–18].

We had previously compared FIT with the urinary biomarker prostaglandin E-major urinary metabolite (PGE-MUM) [19]. The results of that study showed that disease duration affected the correlation between FIT concentrations and endoscopic scores; thus, the shorter the disease duration, the stronger the correlation. In contrast, PGE-MUM showed a high correlation with endoscopic activity during long-term disease. Thus, UC disease duration may affect the accuracy of each biomarker, and these effects may differ across biomarkers. However, though there are many reports investigating the relationship between endoscopic score and FC and FIT, the effect of disease duration on FC, which is a biomarker often used in clinical practice, has not yet been reported [5, 7, 8, 12, 13].

Therefore, this study examined the effect of UC disease duration on FC in comparison with FIT, which has already been shown to be particularly useful for evaluating short-term disease.

## Methods

### Patients

Patients with UC who were treated at Hamamatsu University School of Medicine between February 2019 and December 2021 were included. A total of 128 colonoscopic examinations and biomarker measurements were performed on 87 patients with UC. The enrolled patients were diagnosed with UC according to recent guidelines by evaluation of the clinical course, typical symptoms, and endoscopic and histological findings [20]. Patients with other IBDs, including Crohn’s disease (CD), Behçet’s disease, and indeterminate colitis, were excluded. Patients with malignant tumors such as colorectal cancer were also excluded. This study aimed to assess the association of colonoscopy with the endoscopic score of the entire colon in UC patients with a history of colorectal surgery and those who did not undergo total colonoscopy.

### Study design

This was a single-center prospective cross-sectional study that aimed to investigate the effect of UC disease duration on FC and FIT concentrations. The primary outcome measure in this study was the correlation of endoscopic scores with FC and FIT concentrations in relation to disease duration. Secondary endpoints were evaluated using the receiver operating characteristic analysis of FC and FIT to predict mucosal healing for each disease period.

**Fig. 1:**
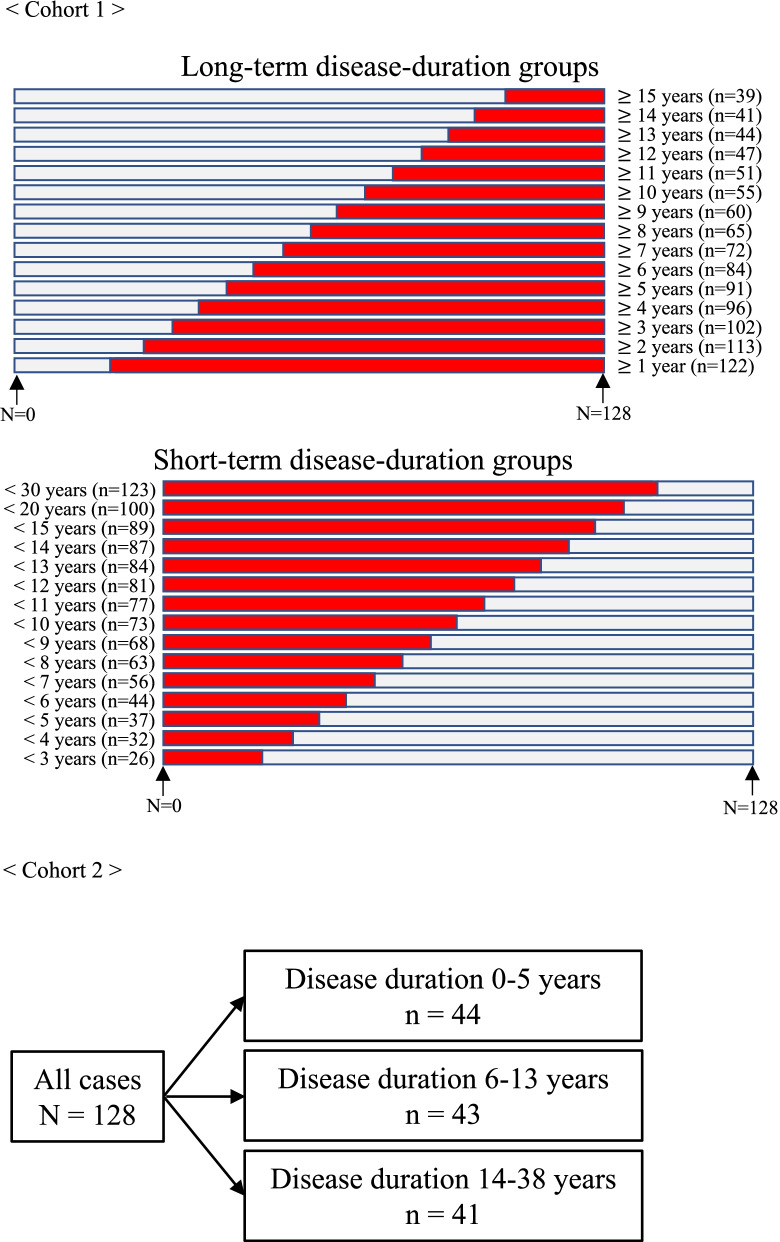
Study flow diagram. Two cohorts were evaluated in the present study. In Cohort 1, patients with long- and short-term disease durations were extracted and analyzed according to disease duration. In Cohort 2, all cases were divided into three groups in the order of disease duration such that the number of cases in each group was almost the same, and analysis was performed in these three groups

In this study, analysis was performed in cohorts 1 and 2, defined according to disease duration (Fig. 1). In Cohort 1, the cases were divided into short- and long-term disease-duration groups to adjust the number of people in each group as evenly as possible. In Cohort 2, the cases were divided into groups with disease durations of 0–5 years (n = 44), 6–13 years (n = 43), and 14–38 years (n = 41), such that the sample size of each group was almost the same, and analyses and comparisons were performed.

### Disease assessment

At the time of enrollment, patients with UC were evaluated using the clinical activity index (CAI) and endoscopic scores. The CAI was assessed using the Rachmilewitz index [21]. Before colonoscopic examinations, patients underwent preparation with a polyethylene glycol-based electrolyte solution. The endoscopic score of the most severe lesion was evaluated using the Mayo endoscopic subscore (MES) and ulcerative colitis endoscopic index of severity (UCEIS). The sum of MES was also scored to evaluate the endoscopic activity of the entire colon [22–24]. MES was evaluated according to the following criteria: 0, normal or inactive disease; 1, mild disease with erythema, decreased vascular pattern, and mild friability; 2, a moderate disease with marked erythema, absence of vascular patterns, friability, and erosions; and 3, severe disease with spontaneous bleeding and ulceration [22]. In this study, only an MES of 0 or 1 was considered to indicate mucosal healing. The UCEIS was calculated as the sum of three descriptors: vascular pattern (score 0–2), erosions and ulcers (score 0–3), and bleeding (score 0–3) [23]. MES was evaluated in five segments, including ascending, transverse, descending, sigmoid colon, and rectum, and the sum of these was calculated as the sum of the MES [24].

### Measurements of biomarkers

To avoid the effects of colonoscopic preparation or examination on biomarkers, fecal samples for FC and FIT were collected the day before or after the colonoscopy. For FC measurements, fecal samples were collected in plastic tubes and stored at -20 ℃ until shipment to the laboratory (SRL Inc., Tokyo, Japan). FC measurements were performed with a Phadia 250 immunoanalyzer (HITACHI Ltd., Tokyo, Japan) and the Elia A Calprotectin 2 reagent (Phadia GmbH, Freiburg, Germany) using fluorescence enzyme immunoassay principles. FIT was performed using a stool collection kit (Eiken Chemical, Tokyo, Japan) for fecal sampling. The submitted samples were immediately processed and measured using OC Sensor IO (Eiken Chemical).

### Statistical analysis

Statistical analyses were performed using the SPSS version 27 (IBM Armonk, New York, NY) and EZR (Saitama Medical Center, Jichi Medical University, Saitama, Japan), which is based on R. More precisely, it is a modified version of R commander designed to add statistical functions frequently used in biostatistics [25]. Correlation analyses were performed using Spearman’s rank correlation test. Differences between median values were compared using the Friedman test, Mann–Whitney U test, or Fisher’s exact test. Receiver operating characteristic (ROC) curve analysis was conducted to determine the optimal cutoff value for predicting mucosal healing. Statistical significance was set at *P* < 0.05.

### Ethics statement

The study protocol was reviewed and approved by the Ethics Committee of the Hamamatsu University School of Medicine (number 18–228). This study was conducted in accordance with the principles of good clinical practice in adherence to the Declaration of Helsinki. All the enrolled patients provided written informed consent to participate in the study.

## Results

### Patient characteristics

One hundred twenty-eight colonoscopies and fecal biomarker measurements were performed in the patients with UC enrolled in this study, and the baseline characteristics are shown in Table 1. The median patient age and disease duration were 48 and 8 years, respectively. The study included 77 men and 51 women. The endoscopic assessments showed an MES of 0, 1, 2, and 3 in 51, 45, 30, and two cases, respectively. The median UCEIS and sum of MES were both 1. The median FC and FIT concentrations were 421 µg/g and 50 ng/mL, respectively.

**Table 1:** Patient characteristics

Characteristics	All N = 128
Age (year), median (IQR)	48 (36–61)
Male/Female, n (%)	77 (60.2)/51 (39.8)
Disease duration (year), median (IQR)	8 (4–17)
Disease extent, n (%)	
Extensive colitis	77 (60.2)
Left-sided colitis	40 (31.2)
Proctitis	11 (8.6)
CAI (Rachmilewitz index), median (IQR)	1 (0–3)
MES, n (%)	
MES 0	51 (39.8)
MES 1	45 (35.2)
MES 2	30 (23.4)
MES 3	2 (1.6)
UCEIS, median (IQR)	1 (0–3)
Sum of MES, median (IQR)	1 (0–3)
FC (µg/g), median (IQR)	421 (76–3408)
FIT (ng/mL), median (IQR)	50 (30–1026)
Medication at study, n (%)	
Oral 5-ASA	88 (68.8)
Suppository steroids	7 (5.5)
Systemic steroids	13 (10.2)
Immunomodulators	35 (27.3)
Advanced therapy	47 (36.7)

*IQR* interquartile range, *CAI* clinical activity index, *MES* Mayo endoscopic subscore, *UCEIS* ulcerative colitis endoscopic index of severity, *FC* fecal calprotectin, *FIT* fecal immunochemical occult blood test, *5-ASA* 5-aminosalicylic acid

### Correlation between MES/UCEIS and fecal biomarkers in the groups with long- and short-term disease durations

The correlations of fecal biomarkers with MES and UCEIS were evaluated in Cohort 1, in which cases were categorized as showing long-term and short-term disease durations. In the analysis of the long-term disease-duration group, the group with a disease duration of 16 years or more was omitted because of their small sample sizes. Similarly, in the analysis of the short-term disease-duration group, the group with a disease duration of ≤ 2 years, which had small sample sizes, was omitted. In addition, the data for groups with disease durations of 20 and 30 years or more were evaluated since the sample sizes of groups with disease durations of 16–38 years or more were small. The participants were grouped as cohort 1 so that the number of enrolled patients increased or decreased evenly according to the disease duration.

**Fig. 2:**
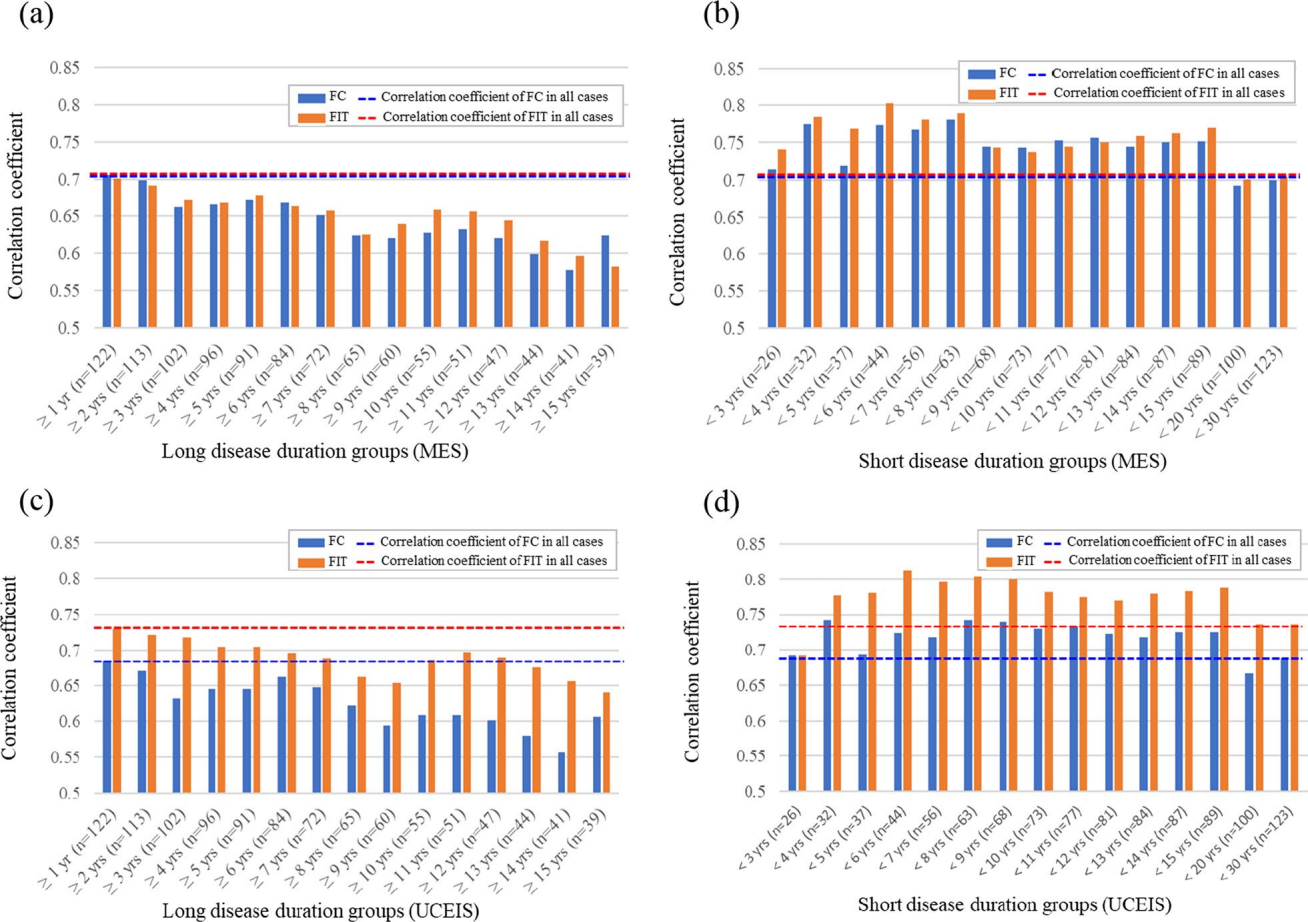
Bar graphs of the correlation coefficients between fecal biomarkers and Mayo endoscopic subscore (MES)/ ulcerative colitis endoscopic index of severity (UCEIS) scores categorized by disease duration. The dark-blue and orange bar graphs represent the fecal calprotectin (FC) and fecal immunochemical occult blood test (FIT) concentrations, respectively. The blue and red dashed lines indicate the correlation coefficients with FC and FIT concentrations in all cases, respectively. Bar graphs of the correlation coefficient between fecal biomarker concentrations and MES in the long-term (**a**) and short-term (**b**) disease-duration groups. Bar graphs of the correlation coefficients between fecal biomarker concentrations and UCEIS in long-term (**c**) and short-term (**d**) disease-duration groups

All groups showed significant correlations between MES and fecal biomarkers, and the bar graphs show the corresponding correlation coefficients. In the analysis of all the cases, the correlation coefficients of MES with FC and FIT concentrations were 0.704 and 0.701, respectively. In the long-term disease duration group, the correlation coefficient between FC concentration and MES in the group with a disease duration of ≥ 1 year was 0.706, which was higher than the correlation coefficient for all cases; however, the correlation coefficients between fecal biomarkers and MES in the other groups were lower than the corresponding correlation coefficients for all cases (Fig. 2a). In the analysis of the short-term disease-duration group, the correlation coefficients between the two biomarkers and MES in the groups with a disease duration of less than 20 and 30 years were lower than the correlation coefficient for all cases, while all other groups showed higher correlation coefficients than those obtained for all cases (Fig. 2b).

Similarly, the correlations between fecal biomarker concentrations and UCEIS were evaluated in the same subgroups. Significant correlations were observed between fecal biomarker concentrations and UCEIS in all group evaluations. The correlation coefficients of UCEIS with FC and FIT concentrations in all cases were 0.685 and 0.738, respectively. In the long-term disease-duration groups, the correlation coefficient between FC concentration and UCEIS was 0.685 in the group with a disease duration of ≥ 1 year, which was equivalent to the correlation coefficient for all cases (Fig. 2c). However, all other groups showed lower correlation coefficients than the corresponding correlation coefficients for all cases. In the analysis of the short-term disease-duration groups, except for the group with a disease duration of < 20 years, the correlation coefficient between FC concentration and UCEIS was higher than that of all cases (Fig. 2d). In addition, the correlation coefficient between FIT concentration and UCEIS was higher than that of all cases, except in the groups with disease duration less than 3, 20, and 30 years.

### Correlation between the sum of MES and fecal biomarkers in the long- and short-term disease-duration groups

**Fig. 3:**
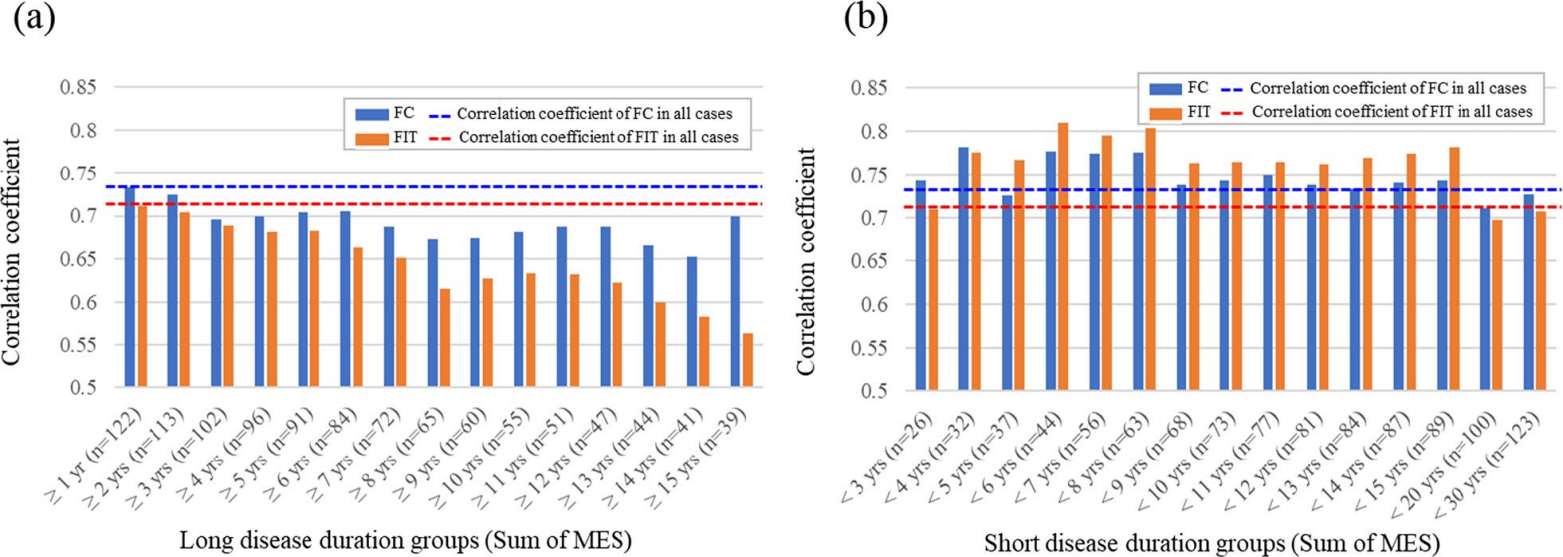
Bar graphs of the correlation coefficient between fecal biomarkers and the sum of Mayo endoscopic subscore (MES) by disease duration. The dark-blue and orange bar graphs represent the fecal calprotectin (FC) and fecal immunochemical occult blood test (FIT) concentrations, respectively. The blue and red dashed lines indicate the correlation coefficients with the FC and FIT concentrations in all cases, respectively. Bar graphs of the correlation coefficients with fecal biomarkers and the sum of MES in the long-term (**a**) and short-term (**b**) disease-duration groups

The correlation between fecal biomarkers and the sum of MES was evaluated in the same subgroups described above. All the subgroups showed a significant correlation between biomarkers and the sum of MES. The correlation coefficients of the sum of MES with FC and FIT concentrations in all the cases were 0.729 and 0.712, respectively. In the analysis of the long-term disease-duration group, the correlation coefficients of all subgroups were lower than that of all cases (Fig. 3a). In the analysis of the short-term disease-duration group, the correlation coefficients of all groups except for FC in the groups with disease duration < 5, < 20, and < 30 years and FIT in the groups with disease duration < 3, < 20, and < 30 years was higher than that in all cases (Fig. 3b).

### Correlation between endoscopic scores and fecal biomarker concentrations in the three groups categorized by disease duration

In Cohort 2, all cases were arranged in order of disease duration and divided into three groups of size: disease duration 0–5 years, 6–13 years, and 14–38 years. A comparison of the patient characteristics at enrollment in the three groups showed a significant difference only in disease duration (*P* < 0.001; Table 2). Correlations between fecal biomarkers and endoscopic scores were evaluated in the three groups (Table 3). Fecal biomarkers and endoscopic scores showed significant correlations in all three groups. In assessments of the correlation between FC and the sum of MES, the correlation coefficient in the disease duration 6–13 group was the highest. Except for this finding, the correlation coefficient between fecal biomarkers and the endoscopic score tended to decrease as the disease duration increased.

**Table 2:** Patient characteristics of the three groups according to disease duration

	Disease duration 0–5 years n = 44	Disease duration 6–13 years n = 43	Disease duration 14–38 years n = 41	*P* value
Age (year), median (IQR)	44.5 (31.8–60.3)	44 (28–62)	51 (43–60)	0.162
Male/Female, n (%)	30 (68.2)/14 (31.8)	26 (60.5)/17 (39.5)	21 (51.2)/20 (48.8)	0.279
Disease duration (year), median (IQR)	2 (1–4)	8 (6–10)	25 (18–28)	< 0.001
Disease extent, n (%)				
Extensive colitis	25 (56.8)	32 (74.4)	20 (48.8)	0.085
Left-sided colitis	13 (29.5)	9 (20.9)	18 (43.9)	
Proctitis	6 (13.6)	2 (4.7)	3 (7.3)	
CAI (Rachmilewitz index), median (IQR)	1 (0–4)	1 (0–2)	1 (0–3)	0.398
MES, n (%)				
MES 0	15 (34.1)	17 (39.5)	19 (46.3)	0.683
MES 1	15 (34.1)	18 (41.9)	12 (29.3)	
MES 2	13 (29.5)	8 (18.6)	9 (22.0)	
MES 3	1 (2.3)	0 (0.0)	1 (2.4)	
UCEIS, median (IQR)	1 (0–3)	2 (0–2)	1 (0–3)	0.662
Sum of MES, median (IQR)	1 (0–4)	1 (0–3)	1 (0–2)	0.576
FC (µg/g), median (IQR)	628 (169–3515)	428 (89–4420)	223 (46–1060)	0.253
FIT (ng/mL), median (IQR)	50 (30–1145)	125 (30–808)	30 (30–699)	0.767
Medication at study, n (%)				
Oral 5-ASA	25 (56.8)	30 (69.8)	33 (80.5)	0.062
Suppository steroids	4 (9.1)	1 (2.3)	2 (4.9)	0.374
Systemic steroids	5 (11.4)	4 (9.3)	4 (9.8)	0.946
Immunomodulators	11 (25.0)	17 (39.5)	7 (17.1)	0.063
Advanced therapy	15 (34.1)	21 (48.8)	11 (26.8)	0.102

*IQR* interquartile range, *CAI* clinical activity index, *MES* Mayo endoscopic subscore, *UCEIS* ulcerative colitis endoscopic index of severity, *FC* fecal calprotectin, *FIT* fecal immunochemical occult blood test, *5-ASA* 5-aminosalicylic acid

**Table 3:** Correlation between endoscopic scores and fecal biomarkers of the three groups according to disease duration

	Disease duration 0–5 years n = 44		Disease duration 6–13 years n = 43		Disease duration 14–38 years n = 41	
	*P* value	r	*P* value	r	*P* value	r
FC						
MES	< 0.001	0.773	< 0.001	0.764	< 0.001	0.577
UCEIS	< 0.001	0.776	< 0.001	0.725	< 0.001	0.653
Sum of MES	< 0.001	0.724	< 0.001	0.746	< 0.001	0.557
FIT						
MES	< 0.001	0.803	< 0.001	0.742	< 0.001	0.597
UCEIS	< 0.001	0.81	< 0.001	0.745	< 0.001	0.582
Sum of MES	< 0.001	0.813	< 0.001	0.757	< 0.001	0.657

*r* correlation coefficient, *FC* fecal calprotectin, *MES* Mayo endoscopic subscore, *UCEIS* ulcerative colitis endoscopic index of severity, *FIT* fecal immunochemical occult blood test

### Receiver operating characteristic analysis for prediction of mucosal healing in the three groups categorized by disease duration

**Fig. 4:**
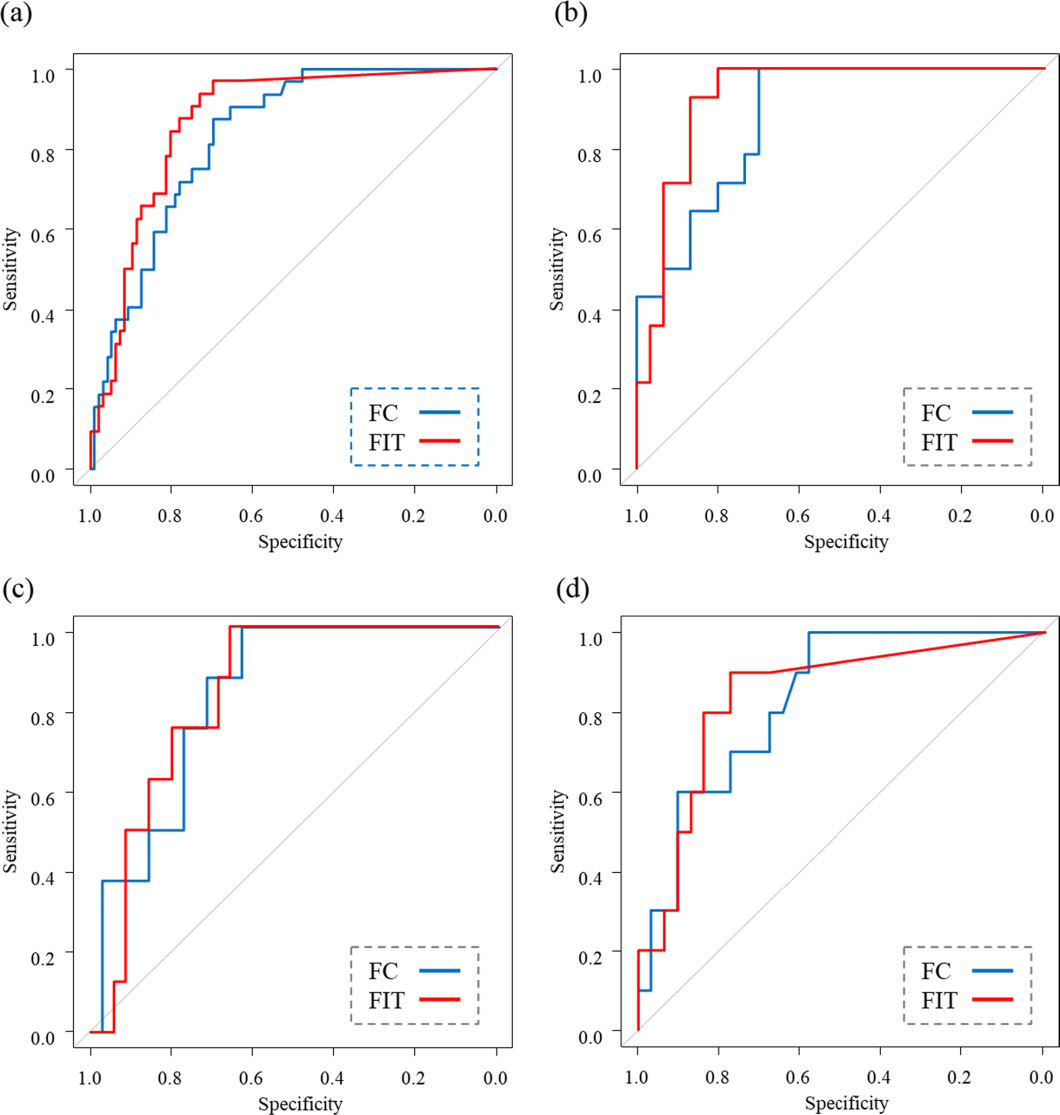
Receiver operating characteristic (ROC) curve analysis for predicting mucosal healing in the three groups according to disease duration. ROC curve of fecal calprotectin (FC) and fecal immunochemical occult blood test (FIT) in all cases (**a**). ROC curves of FC and FIT in the groups of disease duration 0–5 years (**b**), 6–12 years (**c**), and 13–38 years (**d**)

Finally, ROC analysis was performed to calculate the cutoff value of fecal biomarker concentrations for predicting mucosal healing (MES 0 or 1) in the three groups (Table 4) (Fig. 4). The cutoff values of FC and FIT concentrations in all cases were 611 µg/g and 124 ng/mL, respectively. The area under the curve (AUC) values for FC and FIT concentrations were 0.834 (95% confidence interval (95% CI): 0.764–0.905) and 0.868 (95% CI 0.804–0.933), respectively, with no significant difference between these AUCs. In the ROC analysis of the FC concentration for predicting mucosal healing, the AUC of the disease duration 0–5 years group was as high as 0.879 (95% CI 0.780–0.977). The AUCs of FC concentration in the disease duration 5–12 years and 13–38 years groups were 0.832 (95% CI 0.704–0.960) and 0.831 (95% CI 0.701–0.960), respectively, which were almost the same. In the ROC analysis of the FIT concentration prediction for mucosal healing, the AUC of the disease duration 0–4 years group was as high as 0.929 (95% CI 0.854–1.000). The AUCs of the disease duration 5–12 years and 13–38 years groups were about the same at 0.836 (95% CI 0.713–0.959) and 0.840 (95% CI 0.698–0.983), respectively.

**Table 4:** Receiver operating characteristic curve analysis for predicting mucosal healing in the three groups according to disease duration

		Cut-off value	Sensitivity	Specificity	AUC [95% CI]	*P* value
All	FC	611	87.5	69.8	0.834 [0.764–0.905]	0.400
(N = 128)	FIT	124	93.8	72.9	0.868 [0.804–0.933]	
Disease duration 0–5 years	FC	611	100	70	0.879 [0.780–0.977]	0.340
(n = 44)	FIT	96	100	80	0.929 [0.854–1.000]	
Disease duration 6–12 years	FC	516	100	62.9	0.832 [0.704–0.960]	0.940
(n = 43)	FIT	159	100	65.7	0.836 [0.713–0.959]	
Disease duration 13–38 years	FC	171	100	58.1	0.831 [0.701–0.960]	0.920
(n = 41)	FIT	124	90	77.4	0.840 [0.698–0.983]	

*AUC* area under the curve, *95% CI* 95% confidence interval, *FC* fecal calprotectin, *MES* Mayo endoscopic subscore, *UCEIS* ulcerative colitis endoscopic index of severity, *FIT* fecal immunochemical occult blood test

## Discussion

Mucosal healing is an important therapeutic goal in the management of UC, and biomarkers for the evaluation of mucosal healing have been widely used in clinical practice [26]. Endoscopic severity has been reported to predict the prognosis of UC, and the achievement of mucosal healing has been recognized to be important [2, 3]. For this reason, although biomarkers have been recognized to predict the prognosis of UC because they reflect endoscopic activity, recent studies have suggested that biomarker values themselves may also have prognostic significance [12–15]. Furthermore, the prognosis of UC has been reported to be improved by strengthening treatment in cases with elevated biomarker levels; therefore, measurement of biomarkers can also contribute to improvements in the clinical management of UC [27, 28]. Thus, perceptions of biomarker measurements have changed progressively with accumulating evidence describing various aspects of biomarkers. While fecal biomarkers such as FC and FIT were initially thought to be the primary biomarkers, LRG, a serum biomarker, has recently gained more importance in clinical practice, and the role of biomarker measurement has expanded in the management of UC [16–18]. C-reactive protein (CRP), a marker that reflects acute inflammation in various diseases, has also been shown to reflect disease activity in IBD. In addition, various other markers that are not widely used in current clinical practice, including fecal lactoferrin, PGE-MUM, and miRNA, have been investigated [29–32]. Each biomarker has its own characteristics, and an understanding of these characteristics is important when making measurements.

For example, from the viewpoint of sample collection, while blood and urine can be collected in a relatively homogenous state, the collection of fecal samples and the accuracy of the tests using these samples can be influenced by the collection site and the condition of the stool. Additionally, patient hesitation is another factor to consider when a stool sample has to be collected and brought to the hospital. In terms of biomarker accuracy, FC has been reported to more accurately reflect endoscopic severity than CRP [33], and low CRP levels do not necessarily reflect the absence of endoscopic activity [34]. However, our previous report, which evaluated the relationships between biomarkers and the endoscopic score of the entire colon, showed that CRP might strongly reflect the activity of UC in cases showing endoscopic activity [35]. Thus, considering the variety of biomarkers for UC and the individual characteristics of each marker, as well as the effects of different factors on their accuracy, we aimed to investigate the effect of disease duration on FC and FIT, which are two commonly used biomarkers.

In a previous study of biomarkers in UC, we reported a strong correlation between FIT concentration and endoscopic scores, especially in short-term disease-duration groups, and showed that UC disease duration could affect the accuracy of FIT evaluation [19]. We suspected that FIT represents the amount of bleeding from the intestinal tract due to inflammation of UC and that the amount of bleeding decreases due to scarring over longer disease durations. In contrast, PGE-MUM, a urinary biomarker of UC, tended to show a strong correlation even in long-term disease. On the basis of the results of these FIT and PGE-MUM analyses, we suspected that each biomarker might be affected differently by disease duration. Although FC is widely used in clinical practice for UC as well as in clinical trials, the effects of disease duration on UC have not been investigated. In this study, participants were grouped by disease duration, and the correlation and ROC analyses were performed. For FIT, the correlation coefficient with the endoscopic score tended to be lower in the long-term disease-duration group than in all cases and higher in the short-term disease-duration group than in all cases. This result was similar to the findings of our previous report [19]. For FC, the correlation coefficients with the endoscopic score tended to be lower in the long-term disease-duration groups than in all cases and higher in the short-term disease-duration groups than in all cases. FC showed similar results as FIT, indicating that the shorter the disease duration, the more accurate it may be. Nonetheless, the results of these analyses do not provide the necessary clarity to precisely define the duration of a “long” or “short” disease course. These terms are based on the observed tendency of the data. For accurate disease duration, additional cases need to be investigated first.

Although the mechanism underlying the greater accuracy of FC in the short-term disease-duration group is unclear, we hypothesized that fibrosis of the intestinal tract caused by prolonged disease duration might impede the normal expression of neutrophils in the intestinal tract. As a result, long-term disease duration affects FC concentration, which reflects the presence of neutrophils in the intestinal tract. In fact, in the comparisons among the groups in Cohort 2, although the median FC concentration was not significantly different, it was lower in the long-term disease-duration group (Table 2). In addition, the risk of carcinogenesis is known to increase with prolonged UC duration; Hata et al. reported that the cumulative incidence of invasive cancer in UC was 0.5% at 10 years, 4.1% at 20 years, and 6.1% at 30 years [36]. As a mechanism of inflammatory carcinogenesis in such cases of UC, the oxidative stress associated with inflammation may cause damage to the DNA of the mucous membrane and induce carcinogenesis through dysplasia by activation of the carcinogenic gene and suppression of the carcinogenic suppressor gene [37, 38]. We suspect that DNA damage via this mechanism may also alter the inflammatory cytokine profile, like the apoptosis signal pathway due to DNA damage, in the intestinal tract and may affect FC, which reflects neutrophils in the intestinal tract [39].

Furthermore, in the ROC analysis for predicting mucosal healing, both FC and FIT showed high AUCs, especially in the group with a disease duration of 0–4 years. The other two groups showed lower AUCs and had similar AUCs for both FC and FIT concentrations, respectively. As described above, in the ROC analysis, the effects of disease duration were similar between FC and FIT concentrations, and this effect may contribute to a more accurate prediction of mucosal healing in shorter disease durations. To date, no reports have investigated the effect of disease duration in UC on FC. A previous study evaluated the relationship between FC and disease duration for CD by dividing patients into groups with disease duration less than 10 years and ten years or longer. The authors reported that disease duration did not affect the diagnostic usefulness of FC [40].

This study had several limitations. The first limitation is the small number of participants since this was a single-center study. Although the analysis was performed in groups representing each disease duration in Cohort 2, a larger sample size per group would result in more accurate analysis. Additionally, a larger overall sample size would facilitate more detailed evaluations of disease duration divisions by increasing the number of subgroups. Second, in Cohort 1, the sample size differed depending on the subgroup for each disease duration. If the correlation coefficients are to be compared, it is desirable that the sample sizes are the same; however, in this analysis, the number of patients to be analyzed varied from 39 to 122 and 26 to 123 in the long-and short-term disease-duration groups, respectively. However, regarding the analysis of the short-term disease-duration groups, the short-term duration groups had high correlation coefficients despite the small sample size, and this result further supported the possibility that the correlation coefficient was high in the short-term disease-duration group. Third, cases without a total colonoscopy were excluded. Endoscopic examination in patients with severe IBD is associated with the risk of serious complications such as perforation [41, 42]. In addition, the evaluation of severe UC may have been insufficient because it was evaluated by a short colonoscopy, and colonoscopic examination was omitted in consideration of the patient's burden.

## Conclusions

As a biomarker in UC, FC may be affected by disease duration similar to FIT. It was considered particularly useful in UC patients with short disease duration.

## Data Availability

The datasets generated and/or analyzed during the current study are not publicly available due to confidentiality of information but are available from the corresponding author on reasonable request.

## Abbreviations

95% CI: 95% Confidence interval
AUC: Area under the curve
CAI: Clinical activity index
CD: Crohn's disease
CRP: C-reactive protein
FC: Fecal calprotectin
FIT: Fecal immunochemical occult blood test
IBD: Inflammatory bowel disease
LRG: Leucine-rich alpha 2 glycoprotein
MES: Mayo endoscopic subscore
PGE-MUM: Prostaglandin E-major urinary metabolite
ROC: Receiver operating characteristic
UC: Ulcerative colitis
UCEIS: Ulcerative colitis endoscopic index of severity
